# Spatial-temporal trends in the risk of illicit drug toxicity death in British Columbia

**DOI:** 10.1186/s12889-022-14586-8

**Published:** 2022-11-18

**Authors:** Kevin Hu, Brian Klinkenberg, Wen Qi Gan, Amanda K. Slaunwhite

**Affiliations:** 1 British Columbia Centre for Disease Control, 655 West 12th Avenue, Vancouver, BC V5Z 4R4 Canada; 2 Department of Geography, University of British Columbia, Vancouver, BC Canada; 3 School of Population and Public Health, University of British Columbia, Vancouver, BC Canada

**Keywords:** Overdose, Harm reduction, Healthcare access, Generalized additive models, GIS

## Abstract

**Abstract:**

**Background:**

Illicit drug poisoning (overdose) continues to be an important public health problem with overdose-related deaths currently recorded at an unprecedented level. Understanding the geographic variations in fatal overdose mortality is necessary to avoid disproportionate risk resulting from service access inequity.

**Methods:**

We estimated the odds of fatal overdose per event from all cases captured by the overdose surveillance system in British Columbia (2015 - 2018), using both conventional logistic regression and Generalized Additive Models (GAM). The results of GAM were mapped to identify spatial-temporal trends in the risk of fatal overdose.

**Results:**

We found that the odds of fatal overdose were about 30% higher in rural areas than in large urban centers, with some regions reporting odds 50% higher than others. Temporal variations in fatal overdose revealed an increasing trend over the entire province. However, the increase occurred earlier and faster in the Interior and Northern regions.

**Conclusion:**

Rural areas were disproportionately affected by fatal overdose; lack of access to harm reduction services may partly explain the elevated risk in these areas.

**Supplementary Information:**

The online version contains supplementary material available at 10.1186/s12889-022-14586-8.

## Background

Illicit drug poisoning (overdose) continues to be an important public health issue in North America. The economic burden of opioid use disorder and fatal overdose in the United States was estimated to be 1.02 trillion dollars in 2017 alone [1]. In Canada, the province of British Columbia (BC) experienced some of the highest rates of illicit drug-related overdose, with the Provincial Health Officer declaring a provincial public health emergency in 2016. Illicit drug overdose is now the foremost cause of unnatural deaths in BC, exceeding all other causes combined [2].

In BC, the primary cause of the overdose crisis has been contamination of the illicit drug supply with fentanyl. Fentanyl is a highly potent synthetic opioid and was detected in about 90% of the 1550 fatal overdose cases in 2018, jumping from 30% (of 529 cases) in 2015 and 5% (of 270 cases) in 2012 [2]. Harm reduction is an essential component of BC’s overdose response [3], and the authorities have upscaled multiple programs to expand access to Opioid Agonist Therapy (OAT), Take-Home Naloxone (THN), and harm reduction sites (including supervised consumption sites and overdose prevention sites). OAT is an opioid use disorder treatment that prescribes slow-release opioid medications to patients for managing withdrawal symptoms. As patients rarely overdose on these prescribed medications, continuous use of OAT prevents overdose occurrence. Naloxone is an antagonist to opioids that can reverse an overdose. Persons who use substances can obtain portable naloxone kits through the THN program at pharmacies or consume substances under supervision at naloxone-equipped harm reduction sites. Thus, THN and harm reduction sites can prevent overdose death during an event [4]. In 20 months after the 2016 public health emergency declaration, the combined impact of these three interventions was estimated to be 3030 fatal events prevented, whereas 2177 deaths were observed [5]. Given these interventions have been effective at reducing mortality, future resource allocation is more complicated than ever because the distribution of fatal overdoses is now affected by influential interventions in addition to endogenous risk factors. Thus, understanding the spatial variations in fatal overdose risk is necessary to avoid disproportionate risk resulting from service access inequity.

Relevant recent attempts focused on the rural-urban difference in fatal overdose risk and are mostly limited to ecological studies. For example, both Monnat [6] and Hedegaard et al. [7] conducted a county-level study and found that drug overdose mortality rates were higher in urban than in rural counties in the United States, while a descriptive study in BC [8] found rates did not vary much by geographic regions.

The prevailing evidence on the spatial distribution of fatal overdose is insufficient to guide resource allocation. Besides ecological fallacy, these studies (e.g., [6–8]) do not account for repeated overdose events within person. However, this missing consideration is necessary for precise allocations of interventions with different mechanics. For example, if high mortality rates in some regions were mainly driven by frequent overdose occurrence instead of a higher chance of death per event, the addition of more harm reduction sites to these areas (i.e., where people who survived multiple events are relatively common) could be an inefficient use of resources. Thus, previous work lacks the detail to support the optimization of the combined intervention approach in BC.

We examined the spatial variations in fatal overdose risk from an event-based perspective to inform decision-makers on where help is the most needed and what type of interventions would be suitable for particular regions. We first conducted a conventional multiple logistic regression analysis to identify the direction of rural-urban difference in the likelihood of fatal overdose per event. We then proceeded to detect the high-risk areas using a novel spatial modeling approach. In light of the changing landscape of the crisis, we finally visualized the temporal trend in fatal risk by snapshots of the spatial pattern at time intervals.

## Method

### Data and case definition

Our analyses used secondary data contained in the BC Provincial Overdose Cohort hosted at the BC Centre for Disease Control. The data originated from a population-based cohort study that includes people who experienced at least one non-fatal or fatal overdose event in BC between Jan 1, 2015, and Dec 31, 2018. Overdose events were identified from linked (at person-level) administrative data sources such as ambulance service reports, hospitals, BC Coroner’s Service, Vital Statistics, prescription medication dispensing, and provincial correctional institutions. The linkage strategy and source-specific definitions of overdose are described in detail elsewhere [8]. In brief, the captured events are a) ambulance-attended event records that naloxone was administered or that the impression code is overdose-related, b) primary care records (e.g., emergency department visit, hospital discharge) with an overdose-related International Classification of Diseases code, or c) illicit drug toxicity deaths reported by the BC Coroner’s Service or Vital Statistics. Intra-person records that are no more than 24 hours apart were collapsed as one event. Healthcare utilization records since Jan 1, 2010, are also available in the Cohort.

### Exposure and outcome

We examined the association between event location environment and the likelihood of an overdose event being fatal. We hypothesized that events in rural areas may have elevated fatal risk and used the level of urbanicity at the overdose location as the exposure measure (to rurality). Overdose locations were determined from the postal code of injury in ambulance or mortality records. The postal codes’ urbanicity levels were then assigned based on the “Population Centre” variable in the 2016 Canadian census [9], which classifies all areas in the province into four groups based on population size: 1) large urban population centers have a population of 100,000 or more, 2) the medium class is between 30,000 and 99,999, 3) small population centers have 1000 to 29,999 persons, and 4) all areas outside of population centers are categorized as rural. Approximately 13% of BC’s five-million population reside in rural areas whereas 66, 9 and 12% live in large, medium and small urban centers, respectively.

### Covariates

Age, sex, social deprivation, prior overdose experience, other substance uses, and event location type are included as covariates in the analysis. We selected the covariates using a causal diagram, or directed acyclic graph, to minimize confounding bias [10]. The diagram and explanation are presented in the Supplementary material. To quantify social deprivation, we used the social component in the Pampalon index [11] that reflects the prevalence of single-parent families and people living alone at the neighborhood level. The score was employed as a surrogate for the likelihood of using drugs alone – fatal overdose is more likely to occur when using drugs alone than when bystanders are present to help [12]. We assigned the neighborhood scores to persons (and then to events) through home postal codes in the same manner as the derivation of urbanicity. For prior overdose experience, we differentiated a person’s first event from their recurrent ones with a binary indicator and anticipated that having overdose experience may trigger people’s adaptation to alleviate fatal risk in subsequent events. To account for other substance uses, we considered three types of relevant medications: opioids for pain (e.g., prescribed opioids excluding OAT medications), benzodiazepines, and other sedatives (e.g., for sleeping aid). Sedatives and opioids are both respiratory depressants and are known to increase the chance of overdose and death when used together [13]. Three binary indicators, one for each type, are created for having active prescriptions at the time of overdose.

In addition, we included an adjustment for location types (e.g., private residence, public building, outdoor) summarized from record descriptions written by paramedics or the BC Coroner’s Service. Some location types are inherently at high risk of fatal overdose, e.g., abandoned buildings or remote outdoor locations, because the probability of being helped is slim in those places [12]. Predictably, hotspots of fatal risk would be detected mainly around those rare, remote locations if the effect of location types were not removed. Those places are often impractical intervention destinations, so removing their effects is beneficial.

### Statistical analysis

First, we estimated the odds of an overdose being fatal with logistic regression, yielding odds ratios (ORs) for the urbanicity strata and covariates. To account for the correlation between events from the same person, we computed the estimates using the Generalized Estimating Equations (GEE) approach for clustered data proposed by Zeger and Liang [14], adopting an exchangeable working dependence structure.

We further mapped the odds ratios of fatal overdose utilizing the two-dimensional smoothing feature in Generalized Additive Models (GAMs). We retained the same GEE framework and covariate adjustments as the conventional logistic model but substituted the urbanicity term with a smooth function of location coordinates (i.e., replacing a coarse stratification of the event environment with a continuous variable). This smooth function can be pictured as a continuous “surface” of ORs estimated by a GAM.

Applications of modeling spatial variation in disease risks using GAM have been fully described elsewhere [15–17]. In essence, the odds of fatal overdose were modeled as:

1 $$\textrm{logit}\left[P\left({Y}_i=1\right)\right]=S\left({\textrm{e}}_i,{\textrm{n}}_i\right)+{\boldsymbol{\beta} \boldsymbol{Z}}_{\boldsymbol{i}}$$

Where i = 1, …, n are the n^th^ observations, Y is a binary indicator of an event being fatal, **Z** is a vector of covariates, and **β** is a vector of coefficients. S is a smoother function (e.g., a spline term) of the event location coordinates (easting, northing) in a projected coordinate system. It can be interpreted as the proportion of log-odds unexplained by **Z** tied to the location. In other words, exp.(S) is estimated OR at each location adjusted for covariates. The models were fitted using the “mgcv” package [18] in statistical software R, adopting Thin Plate Regression Spline [19] as the smoothing basis.

The resulted distribution of cumulative (over the 4 years) ORs of fatal overdose was visualized as heat maps at the province scale along with 95% Bayesian confidence intervals [20]. To pinpoint at-risk neighborhoods, we repeated the same analysis at the city-scale for Vancouver, Victoria, Kelowna, and Prince George, the largest cities in BC; separate models were fitted to subsets of the data by city. Harm reduction sites were also plotted on the map to assess their impact on the risk pattern. There is no site in rural settings yet, so that no rural communities were chosen for the city-scale analysis.

Finally, we examined the spatial-temporal trends of fatal overdose (at the province scale) by incorporating space and time interaction into the GAM [21], e.g., *S*(*e*, *n*) ∗ *t* where *t* is the number of months between Jan 1, 2015 and when the event happened. Then risk surfaces were mapped every 6 months to resemble the spatial-temporal trends. Similarly, we also included a year variable and its interaction with urbanicity in the logistic model to reflect the temporal aspect. The year variable differentiates periods before and after Jan 1, 2017; as most overdose prevention sites were launched in late 2016, we expect changes in fatal risk during the second half of the study period.

Two sensitivity analyses were performed. First, events with incomplete location information were removed from the data analyses; consequently, we investigated the potential bias arising from this exclusion. As the missing data is mainly in the location type variable, we fitted an adjusted model without the location type variable using almost all events. Then, it was compared to an identical model with data exclusion. Second, we explored the impact of the choice of smoothing basis on the mapping result. Risk surfaces produced by two alternative bases, Duchon splines and Gaussian process (equivalent to kriging), were compared to the presented result.

## Results

### Descriptive analysis

During the study period, 42,711 overdose events (26,835 persons) were identified, and of these, 35,569 (83.3%) events had complete location information and were included in the analysis. The 23,191 persons who survived their first overdose contributed a mean of 1.83 (Standard Deviation: 1.1) person-years of follow-up from the date of the first overdose to death or Dec 31, 2018; the large population center group has slightly longer follow-up time on average (mean: 1.88, SD: 1.1), but the distributions are similar across urbanicity (e.g., mean: 1.73, SD: 1.06 in the rural group).

A comparison of characteristics between fatal and non-fatal overdose events is summarized in Table 1. The proportion of cases from rural areas was higher among fatal overdoses (9.1%) than non-fatal overdoses (5.7%). Also, the two types of events differ notably by age, sex, and being a recurrent event (24.3% of fatal overdoses vs. 42% of non-fatal overdoses). Recurrent overdose was the least prevalent in rural communities (19.9% of rural events compared to 44.1, 36.6, and 23.8% of large, medium, and small urban center events, respectively). Furthermore, location type seems to profoundly impact the likelihood of fatal overdose; deaths in public buildings and healthcare facilities were infrequent.

**Table 1:** The characteristics of fatal and non-fatal overdoses

Variable	Level	All	Fatal Event^1^	
			No	Yes
	Total	35,569	30,957 (87)	4612 (13)
Urbanicity	Large	24,856 (69.9)	21,905 (70.8)	2951 (64)
	Medium	5485 (15.4)	4725 (15.3)	760 (16.5)
	Small	3028 (8.5)	2547 (8.2)	481 (10.4)
	Rural	2200 (6.2)	1780 (5.7)	420 (9.1)
Location Type	Private	17,551 (49.3)	13,733 (44.4)	3818 (82.8)
	Public Buildings	5123 (14.4)	5031 (16.3)	92 (2)
	Healthcare Facilities	811 (2.3)	775 (2.5)	36 (0.8)
	Outdoor	9917 (27.9)	9546 (30.8)	371 (8)
	Others	2167 (6.1)	1872 (6)	295 (6.4)
Recurrent Event	No	21,450 (60.3)	17,958 (58)	3492 (75.7)
	Yes	14,119 (39.7)	12,999 (42)	1120 (24.3)
Age	18–29	10,873 (30.6)	10,019 (32.4)	854 (18.5)
	< 18	783 (2.2)	746 (2.4)	37 (0.8)
	30–49	16,159 (45.4)	14,035 (45.3)	2124 (46.1)
	50–64	6381 (17.9)	5093 (16.5)	1288 (27.9)
	≥65	1373 (3.9)	1064 (3.4)	309 (6.7)
Sex	F	10,078 (28.3)	9098 (29.4)	980 (21.2)
	M	25,491 (71.7)	21,859 (70.6)	3632 (78.8)
Benzodiazepines Prescriptions	No	33,162 (93.2)	28,982 (93.6)	4180 (90.6)
	Yes	2407 (6.8)	1975 (6.4)	432 (9.4)
Other Sedatives Prescriptions	No	24,740 (69.6)	21,640 (69.9)	3100 (67.2)
	Yes	10,829 (30.4)	9317 (30.1)	1512 (32.8)
Opioids for pain Prescriptions	No	33,254 (93.5)	29,073 (93.9)	4181 (90.7)
	Yes	2315 (6.5)	1884 (6.1)	431 (9.3)
Social Deprivation^2^	Q3	4824 (13.6)	4195 (13.6)	629 (13.6)
	Q1	3143 (8.8)	2658 (8.6)	485 (10.5)
	Q2	4966 (14)	4297 (13.9)	669 (14.5)
	Q4	6499 (18.3)	5658 (18.3)	841 (18.2)
	Q5	16,137 (45.4)	14,149 (45.7)	1988 (43.1)
Year	2015–2016	13,374 (37.6)	11,820 (38.2)	1554 (33.7)
	2017–2018	22,195 (62.4)	19,137 (61.8)	3058 (66.3)

^1^Data are n (%)

^2^The deprivation indexes are categorized by quantile, with Q5 being the most deprived. Q3 is selected as the reference level as it includes the average

### Rural-urban differences in fatal overdose risk

We present the multivariable logistic regression results comparing different combinations of adjustment sets in Table 2. All models agree that rural events had significantly higher odds of being fatal than events in large population centers (the reference), and the odds ratio for rural events ranges from 1.59 (Confidence Interval: 1.42, 1.78) unadjusted to 1.31 (CI: 1.07, 1.60) adjusted for all selected covariates. The odds of fatal overdose in medium and small population centers were also higher than the reference but lost significance after full adjustment. As expected, being a recurrent event reduced the likelihood of fatal overdose by about 40% (OR: 0.60, CI: 0.56, 0.65). The location type variable is a strong predictor of fatality odds: events that happened in private places (e.g., home or vehicles) were about 5 or 12 times more likely to be fatal than those that occurred in healthcare facilities or public buildings, respectively. Surprisingly, there was a higher likelihood of fatal overdose in healthcare facilities than in public buildings. One explanation could be that events in healthcare facilities were from people with pre-existing health conditions. Events happened in the second half of the study period are associated with an 39% (OR: 1.39, CI: 1.28, 1.52) increase in odds of being fatal. However, no significant (multiplicative) interaction between urbanicity and year of the event was detected.

**Table 2:** The associations between odds of fatal overdose and the urbanicity of event environment

	Unadjusted			Adjusted								
Variable	OR^1^	95% CI^1^	p-value	OR^2^	95% CI	p-value	OR^3^	95% CI	p-value	OR^4^	95% CI	p-value
**Urbanicity**												
Large	–	–		–	–		–	–		–	–	
Medium	1.14	1.04, 1.24	0.003	1.27	1.10, 1.47	0.001	1.16	1.00, 1.34	0.057	1.14	0.98, 1.32	0.090
Small	1.29	1.16, 1.43	< 0.001	1.22	1.01, 1.47	0.035	1.06	0.88, 1.29	0.5	1.03	0.85, 1.24	0.8
Rural	1.59	1.42, 1.78	< 0.001	1.57	1.29, 1.91	< 0.001	1.36	1.11, 1.66	0.003	1.31	1.07, 1.60	0.009
**Location Type**												
Private	–	–					–	–		–	–	
Public Buildings	0.07	0.06, 0.09	< 0.001				0.08	0.06, 0.09	< 0.001	0.08	0.06, 0.09	< 0.001
Healthcare Facilities	0.18	0.14, 0.25	< 0.001				0.18	0.13, 0.25	< 0.001	0.19	0.14, 0.27	< 0.001
Outdoor	0.15	0.14, 0.17	< 0.001				0.16	0.14, 0.18	< 0.001	0.16	0.15, 0.18	< 0.001
Others	0.58	0.52, 0.66	< 0.001				0.62	0.54, 0.70	< 0.001	0.65	0.57, 0.75	< 0.001
**Recurrent Event**												
No	–	–		–	–					–	–	
Yes	0.51	0.48, 0.55	< 0.001	0.52	0.48, 0.56	< 0.001				0.60	0.56, 0.65	< 0.001
**Age**												
18–29	–	–		–	–		–	–		–	–	
< 18	0.53	0.38, 0.75	< 0.001	0.50	0.36, 0.71	< 0.001	0.57	0.41, 0.81	0.002	0.54	0.38, 0.76	< 0.001
30–49	1.78	1.63, 1.93	< 0.001	1.73	1.59, 1.89	< 0.001	1.72	1.57, 1.88	< 0.001	1.68	1.54, 1.83	< 0.001
50–64	2.92	2.65, 3.21	< 0.001	2.71	2.45, 2.99	< 0.001	2.41	2.17, 2.67	< 0.001	2.30	2.07, 2.54	< 0.001
≥65	3.13	2.70, 3.63	< 0.001	2.76	2.37, 3.22	< 0.001	2.48	2.11, 2.92	< 0.001	2.24	1.91, 2.64	< 0.001
**Sex**												
F	–	–		–	–		–	–		–	–	
M	1.61	1.49, 1.74	< 0.001	1.62	1.50, 1.75	< 0.001	1.77	1.64, 1.92	< 0.001	1.81	1.67, 1.96	< 0.001
**Benzodiazepines Prescriptions**												
No	–	–		–	–		–	–		–	–	
Yes	1.46	1.30, 1.63	< 0.001	1.43	1.26, 1.63	< 0.001	1.37	1.20, 1.57	< 0.001	1.37	1.20, 1.56	< 0.001
**Other Sedatives Prescriptions**												
No	–	–		–	–		–	–		–	–	
Yes	1.19	1.11, 1.28	< 0.001	0.89	0.81, 0.96	0.005	0.86	0.79, 0.94	< 0.001	0.88	0.81, 0.96	0.004
**Opioids for pain Prescriptions**												
No	–	–		–	–		–	–		–	–	
Yes	1.54	1.38, 1.73	< 0.001	1.14	1.00, 1.30	0.054	1.05	0.92, 1.21	0.5	1.04	0.91, 1.19	0.6
**Social Deprivation**												
Q3	–	–		–	–		–	–		–	–	
Q1	1.20	1.05, 1.37	0.008	1.28	1.12, 1.46	< 0.001	1.24	1.08, 1.43	0.002	1.22	1.06, 1.40	0.004
Q2	1.05	0.93, 1.18	0.5	1.08	0.95, 1.22	0.2	1.12	0.98, 1.27	0.092	1.12	0.99, 1.27	0.083
Q4	1.00	0.89, 1.12	> 0.9	0.99	0.88, 1.11	0.9	0.98	0.87, 1.11	0.8	1.00	0.88, 1.12	> 0.9
Q5	0.96	0.87, 1.06	0.4	0.96	0.87, 1.06	0.4	0.97	0.87, 1.07	0.5	1.00	0.90, 1.11	> 0.9
**Year**												
2015–2016	–	–		–	–		–	–		–	–	
2017–2018	1.26	1.19, 1.34	< 0.001	1.40	1.29, 1.52	< 0.001	1.29	1.19, 1.41	< 0.001	1.39	1.28, 1.52	< 0.001
Urbanicity * Year												
Medium * 2017–2018				0.86	0.72, 1.03	0.11	0.91	0.76, 1.10	0.3	0.91	0.75, 1.09	0.3
Small * 2017–2018				1.07	0.86, 1.35	0.5	1.08	0.85, 1.36	0.5	1.05	0.83, 1.33	0.7
Rural * 2017–2018				0.89	0.70, 1.14	0.4	0.92	0.72, 1.19	0.5	0.90	0.70, 1.15	0.4

^1^*OR* Odds Ratio, *CI* Confidence Interval

^2^Adjusted for all covariates except location type

^3^Adjusted for all covariates except prior overdose experience

^4^Adjusted for all covariates

The asterisk symbol = represents the interaction between two variables

### Mapping spatial variations in fatal overdose risk

**Fig. 1:**
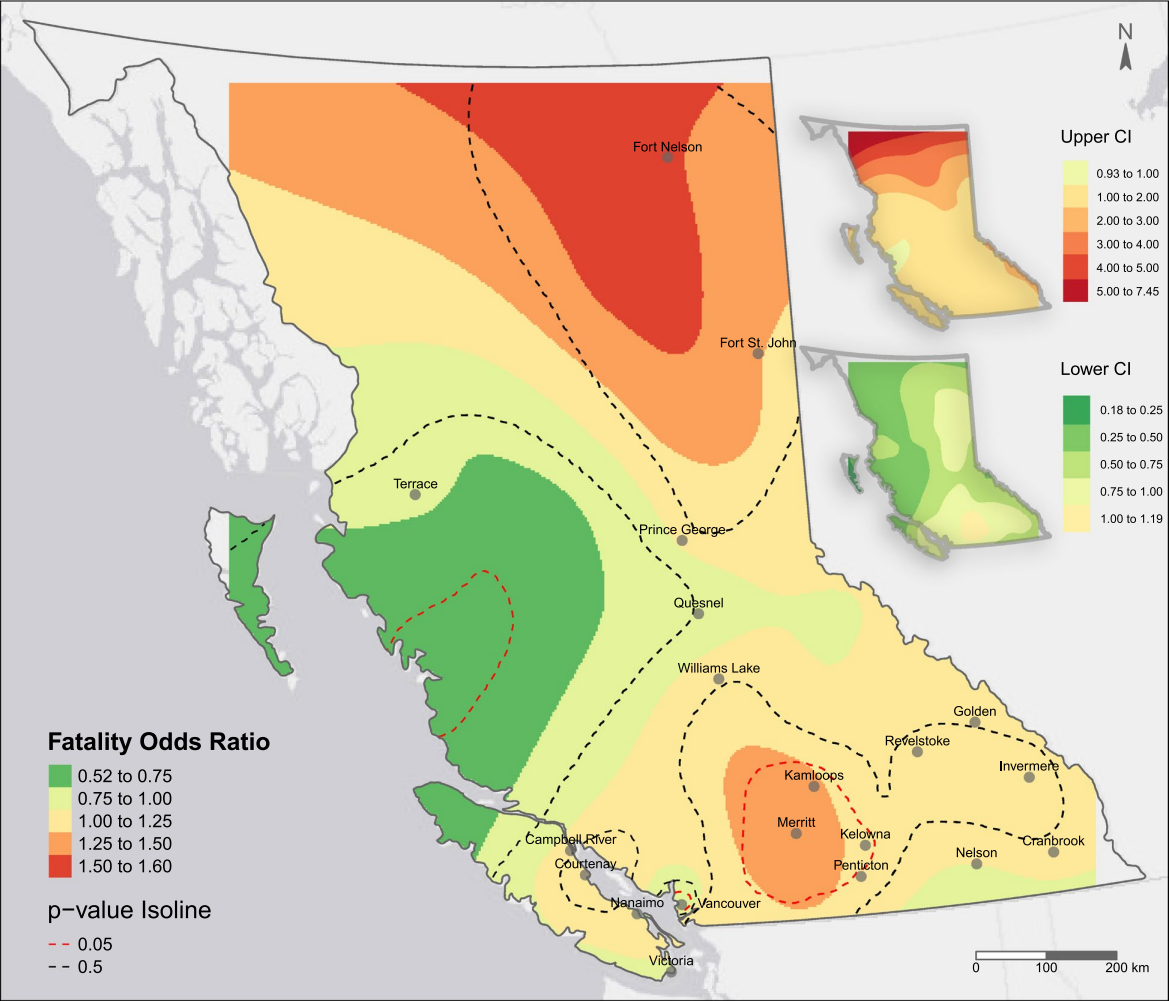
Spatial variations in the adjusted odds of fatal overdose per event with 95% confidence interval (CI). The odds ratios were smoothed within the square bounding box of the data points; thus, the resulting surface does not cover the entire province. Note that excessive extrapolation was kept over uninhabited areas to prevent the re-identification of remote neighborhoods

The spatial pattern of fatal overdose odds per event is visualized as a heatmap in Fig. 1. The estimated effects of the covariates are mostly close to the urbanicity model and not shown here (see supplementary material). A large region centered around Merritt encompassing some major cities in the interior BC (e.g., Kelowna and Kamloops) had odds of fatal overdose approximately 25 to 50% higher than the surrounding areas, whereas Metro Vancouver was a significant cold spot (0.75 ≤ *OR* < 1). The highest estimates appear in the north but are not significant, probably because the number of events is relatively small in those remote areas.

**Fig. 2:**
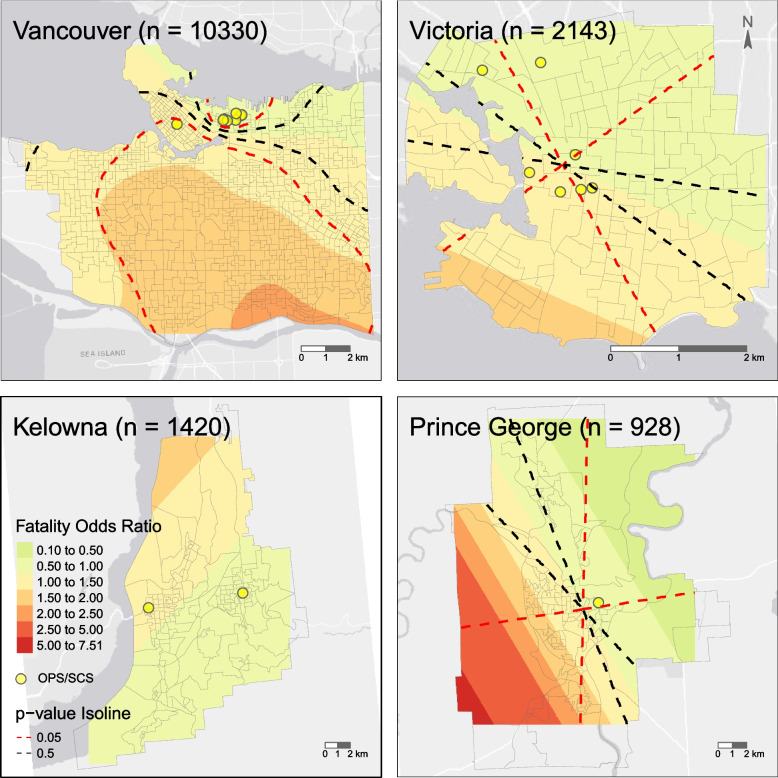
Spatial variations in the adjusted odds of fatal overdose per event in four major cities in British Columbia. Cities without *p*-value isolines had no significant spatial trend. The area units in the background are census dissemination areas; the denser the units, the higher the population density. The yellow dots represent the Overdose Prevention Sites (OPS) or Supervised Consumption Sites (SCS)

The city-scale variations in fatal overdose risk are mapped in Fig. 2. In Vancouver, the cluster of harm reduction sites was found to be a significant cold spot (0.50 ≤ *OR* < 1); contrarily, neighborhoods in the southeast had the highest risk (2.00 ≤ *OR* < 2.5). Although the risk in Victoria and Prince George are not significant at the provincial scale, heterogeneity is present within the city. We observed a gradually increasing trend from north to south in Victoria and that the fatal risk increased rapidly from northeast to southwest in Prince George. Interestingly, the inflection points (with respect to where *OR* = 1) of the spatial trends in these cities, especially in Vancouver, are seemingly in the vicinity of the harm reduction sites, suggesting a possible spatial association between harm reduction sites and low fatal overdose risk.

**Fig. 3:**
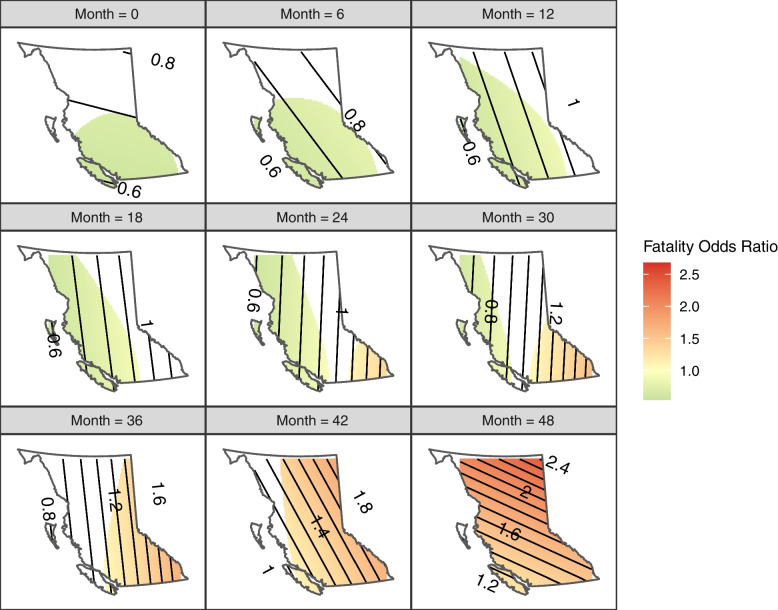
Spatial-temporal variations in estimated fatal overdose odds per event from Jan 1, 2015 to Dec 31, 2018. Areas that are not significant (*p* > 0.05) were not colored, but their odds ratios can be read from the contour lines

The spatial-temporal changes in fatal overdose risk are visualized as snapshots every 6 months from Jan 2015 to Dec 2018 in Fig. 3. In general, the fatal risk increased over time all over BC (month 48 vs. month 0), which is in the same direction as the OR estimate for the year variable in the logistic regression model. More specifically, the magnitude of the elevated risk exhibits a northeast-southwest trend, with the southwest region (e.g., Metro Vancouver and Victoria) having the lowest increase. Considering central BC at the end of 2016 (month 24) as the reference level (where *OR* = 1), the fatal risk was below reference (mostly 0.6 ≤ *OR* ≤ 0.9) over the whole province until the first half of 2016. The spatial trend started to shift after the second half of 2016: the hotspot originated in the east Interior then spread over the province in 2 years. By the end of 2018, the risk in northern BC increased by 2 ~ 2.4 times compared to the reference. Meanwhile, the more urbanized southwest region experienced a relatively slower increase (1.2 ≤ *OR* ≤ 1.6 compared to 0.6 ≤ *OR* ≤ 0.7 at the start of the study period).

### Sensitivity analysis

The impact of excluding events with missing location types on the regression analysis results was minor. The odds ratio estimates for urbanicity levels, age, and opioids for pain prescription were increased slightly without data exclusion; however, the conclusion was not altered. Also, the differences in OR estimates resulting from alternative smoothers in GAMs did not change our interpretations of the spatial patterns; the alternative maps are adequately similar to those presented. Sensitivity analysis results are available in the supplementary material.

## Discussion

### Urbanicity and fatal overdose

We found that the likelihood of fatal overdose increases as the urbanicity of event location becomes more rural. The odds are about 30% higher in rural areas than in large urban centers. Our estimates are conservative, as the prominent, non-manipulatable effect from location types was removed.

The magnitude of this estimated effect of urbanicity might be insubstantial, but the direction of the effect contrasts previous findings. Most of the recent work relevant to the current crisis found higher overdose mortality rates in urban communities [6–8]. As the present and previous work are based on different perspectives, the results are not directly comparable. However, it is noteworthy that previous studies whose conclusions were based on mortality rates (using the general population as the denominator) are subject to limitations associated with estimating the population at risk [22]. In comparison, our estimates were obtained from a representative study population, a large subset of the true population at risk (i.e., people who use substances). Our results are likely less biased and more consistent with the well-known environmental health theories of inequality that postulate that rural communities are disadvantaged in health resources and have socioeconomic stresses leading to adverse health outcomes [23].

Despite our findings supporting a higher likelihood of fatal overdose in rural communities, we do not refute that overdose mortality rates could be higher in urban areas. We observed that recurrent overdoses were two times more prevalent in large urban centers than in rural communities. The damage from this disproportionated prevalence of recurrence could outweigh the relief from advantaged fatal risk per event, leading to an elevated mortality rate in urban settings.

Although the direction of rural-urban difference in terms of the conventional mortality rate measure is unknowable, our event-based findings are practical, suggesting decision-makers can match the mechanism of interventions and local susceptibilities to improve the efficacy of allocation. If an intervention is aimed to reduce overdose recurrence/occurrence (e.g., pharmaceutical alternatives to the toxic drug supply) – of course, all communities will benefit – it would be more efficient in urban areas. On the contrary, the disadvantaged fatal risk per event in rural communities can be mitigated through death-preventing services, e.g., harm reduction sites.

### Spatial-temporal trends in overdose

At the macro scale, we discovered that a region centered at Merritt, a small population center/rural environment, in the Interior BC was the hardest-hit area in terms of fatal risk per event, echoing findings from the urbanicity model. We further observed significant micro-scale spatial heterogeneity in some of the selected cities, demonstrating our approach’s ability to identify at-risk neighborhoods. Interestingly, the city-scale maps also provide insights into the potential factors driving fatal overdose risks. We observed that the turning points (e.g., from below to above reference or vice versa) of the spatial trends often seem related to the locations of harm reduction sites. For example, a significant cold spot encircles the cluster of harm reduction sites in Vancouver Downtown Eastside. Harm reduction sites have been proven to effectively reverse fatal overdoses [4]. It is not surprising that the sites could impact fatal risk – in a *decreasing* direction. However, given the general *increasing* trend from the spatial-temporal pattern, other risk-driving forces besides harm reduction services are present.

We propose that the rising illicit drug toxicity and the lack of access to harm reduction services are the co-drivers of spatial-temporal variations in fatal overdose risk. A recent interview with an illicit drug seller revealed that some persons who use substances actively seek ever-stronger drugs to get high as their tolerance increases over time [24]. This behavior may explain the escalating intended use of fentanyl [25]. Not only that, the demand for ever-stronger drugs is chased by supply; drugs in the illicit market are, in fact, becoming more toxic over time. Recent data [2] indicates that more overdose deaths were associated with extreme fentanyl concentrations or carfentanil (a fentanyl analog for veterinary use on elephants or bears) in 2020 compared to the previous year. Consequently, the rising drug toxicity led to more frequent - also more deadly^1^ - overdoses throughout the province. The increasing fatal risk was undoubtedly lessened by harm reduction services in urban areas; some probable fatal overdoses were converted to non-fatal recurrent events. However, in rural areas with inadequate harm reduction services, it is likely that fatal risks per event will increase as the illicit drug supply becomes more toxic. This is evident in the spatial-temporal pattern we observed: the risk in less urbanized regions in Interior and Northern BC increased earlier and faster than in metropolitan regions in the southwest.

### Limitations

The major limitation of this study is that the data does not cover all the overdose events. Overdose events where a person did not interact with healthcare were not captured. These omitted events were likely non-fatal overdoses averted by THN kits administered by bystanders where health care was not sought, resulting in selection bias. The number of deaths averted by naloxone kits in the first 10 months of 2016 was estimated as about one-third of the observed overdose death [26]. Assuming this proportion applies to our study period, nearly 3% of the total events were averted at home and absent in the data; the resulting selection bias would likely not affect the overall findings.

The omitted events also include those with missing location information. Although the sensitivity analysis suggests that our estimates/findings are stable with and without the missing data exclusion, it is possible that location data was not missing at random but related to cases where the initial healthcare encounter was not at the injury location, e.g., people self-transport to emergency department for treatment. Such events were non-fatal and more common in urban than in rural areas because the chance of having a healthcare facility nearby is higher in the former. Omitting these events might overestimate the mortality risk at large population centers (the reference level), thereby underestimating the OR for rural regions.

Another limitation arises from the assumption embedded in mapping with GAM. The risk surfaces were assumed to be smooth over the bounding box of the data. This assumption may be inappropriate at the province scale; small ups and downs could be informative but diminished after averaging, though this problem can be mitigated by fitting separate surfaces to smaller areas of interest, which has been done to only four major cities. Additional efforts would be required to recover a fully detailed picture of the risks in BC. However, given the observation that only some of these cities had significant micro-scale spatial trends, the information lost in the presented provincial map could still be acceptable.

## Conclusion

In summary, we found that some rural areas experienced high fatal overdose risk per event, potentially due to contamination of the illicit supply and minimal access to harm reduction services. The results of this study suggest that future intervention efforts should prioritize mortality-preventing services in rural/remote settings such as harm reduction sites and safer supply. The findings of this research are critical for need-based planning of harm reduction services in BC, and our spatial modeling approach is applicable elsewhere to locate potential facilities at the neighborhood level. Finally, we should be aware that the findings reflected both the success of interventions in urban regions and the resource deficiency in rural areas.

## Supplementary Information

**Additional file 1.**

## Data Availability

The data used in this paper come from the BC Centre for Disease Control (BCCDC) Provincial Overdose Cohort. Access to the data is strictly regulated, in accordance with the provisions of the Public Health Act. Individuals interested in accessing the data should contact Dr. Amanda Slaunwhite (amanda.slaunwhite@bccdc.ca).

## Abbreviations

BC: British Columbia
CI: Confidence Interval
GAM: Generalized Additive Model
GEE: Generalized Estimating Equation
OAT: Opioid Agonist Therapy
OPS: Overdose Prevention Site
OR: Odds Ratio
SCS: Supervised Consumption Site
SD: Standard Deviation

## Footnotes

1 Naloxone is temporary. Overdose could return if insufficient doses of naloxone are administered.
