# Supplementary material for: Spatial-temporal trends in the risk of illicit drug toxicity death in British Columbia
Source: BMC Public Health. 2022 Nov 18;22:2121. doi: 10.1186/s12889-022-14586-8 (PMC9675064; doi:10.1186/s12889-022-14586-8)
Supplement: Supplementary file 1 — Additional file 1. [file 12889_2022_14586_MOESM1_ESM.docx]

Supplementary material

1. Causal diagram

The causal diagram used in the confounder selection process is presented here, accompanied by a table explaining the connections between factors/variables (see Rothman et al., 2008 for a detailed tutorial). The exposure and outcome are in a d-connected relation (highlighted in blue) after adjustment (variables in squares), meaning no confounder bias if the diagram is correct. The direction of linkages dictates chronological relation and dependency.

D-connected relation suggests that all back-door paths are closed, and only direct paths between the exposure and outcome remain open (Pearl, 2009). For example, in supplementary figure 1, social deprivation is a confounder because it affects both the exposure and outcome (indirectly through other variables). Any path connecting confounders to the outcome (not through the exposure), regardless of the direction of arrows, is a back-door path, such as Link 23 -> 25 -> 15. A path is naturally open if there is no collider (e.g., given X -> Y <- Z, Y is collider as both X and Z point to it), or closed when a collider is present. Adjusting for a collider opens the path (e.g., the dashed paths resulted from adjusting for ‘overdose’ and ‘prior experience’), otherwise conditioning on non-colliders would close the naturally open paths (e.g., Link 25 -> 14).

- 1. Diagram


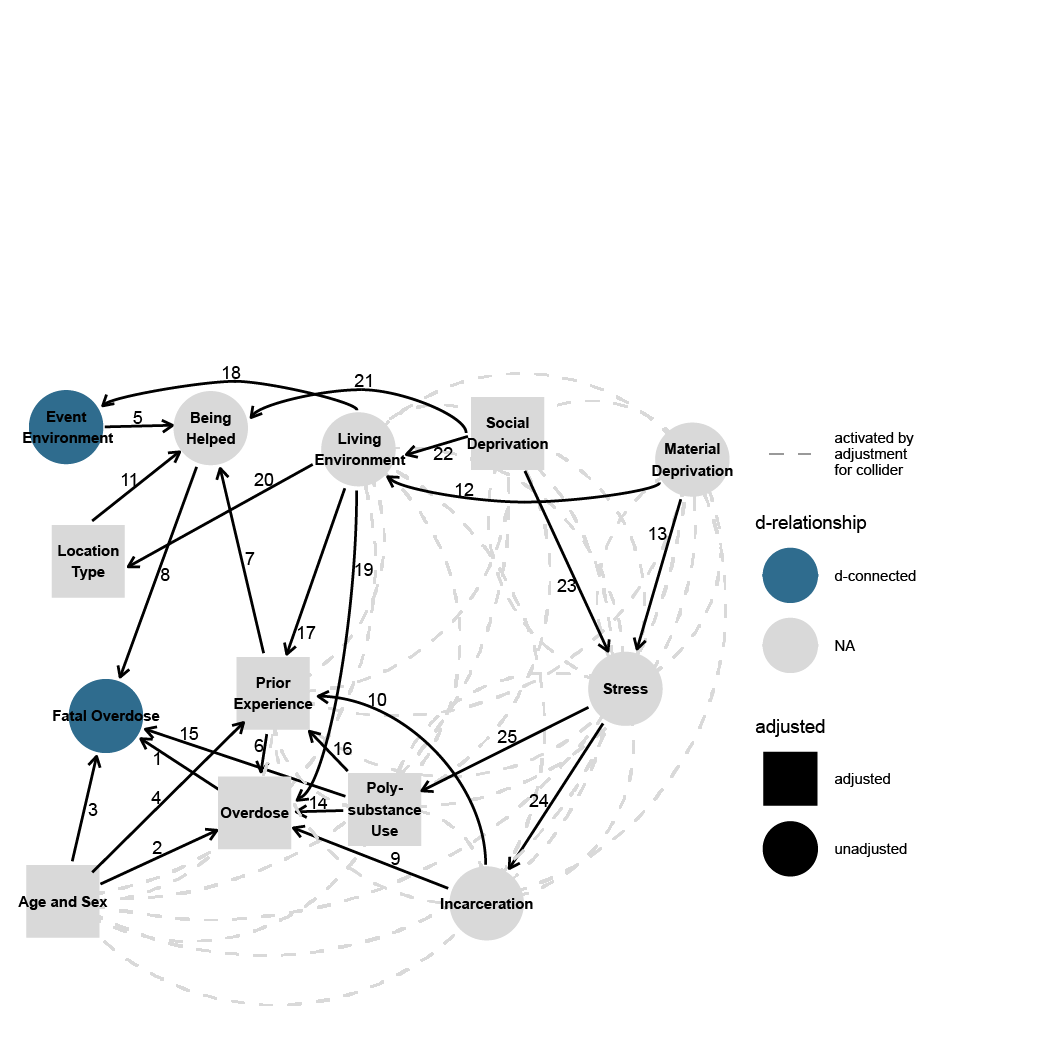


Supplementary figure 1. Causal diagram for the path between event environment and fatal overdose based on literature review.

- 1. Explanation table

Supplementary table 1. Explanations for the links in the causal diagram for the path between event environment and fatal overdose.

| To | From | Link No. | Connection |
| --- | --- | --- | --- |
| Fatal overdose | Overdose | 1 | Overdose occurred before the fatal/non-fatal outcome |
|  | Age and sex | 3 | Individual conditions that are known to affect the outcome of an overdose (Martins et al., 2015) |
|  | Being helped | 8 | Death could be prevented if the overdose event was witnessed |
|  | Poly-substance use | 15 | Using opioids (a depressant) with other sedatives, e.g., benzodiazepines, could double the depressant effect, which also affects the chance of death (White and Irvine, 1999) |
| Being helped | Event environment | 5 | Events happened in urban areas with higher population density could be more likely to be witnessed and helped |
|  | Prior experience | 7 | People with overdose experience may be more likely to use naloxone kits or harm reduction services due to fear of death |
|  | Location type | 11 | Events that happened in public buildings or outside are more likely to be witnessed and helped compared to those in private residences |
|  | Social deprivation | 21 | Socially deprived people may tend to use drugs alone, reducing the chance of being witnessed |
| Overdose | Age and sex | 2 | Individual conditions that are known to affect overdose occurrence |
|  | Prior experience | 6 | People with overdose experience may use services, e.g., OAT, affecting the chance of having recurrent events |
|  | Incarceration | 9 | Recent releases from prison are associated with elevated overdose risk (Bukten et al., 2017) |
|  | Poly-substance use | 14 | Similar to Link 15, Poly-substance use could also affect the likelihood of overdose (Kerr et al., 2007) |
|  | Living environment | 19 | The living environment determines access to healthcare and the availability/prevalence of illicit drug supply. A specific drug that is prevalent in a particular region could be more toxic than the prevalent drugs in other places (Amlani et al., 2015), leading to spatial variations in overdose occurrence. |
| Living environment | Material deprivation | 12 | People that are materially deprived tend to live in low-income neighborhoods |
|  | Social deprivation | 22 | People that are socially deprived tend to live in places such as single room apartments |
| Stress | Material deprivation | 13 | Economic burden creates stress |
|  | Social deprivation | 23 | Social isolation creates stress |
| Event location | Living environment | 18 | Events may tend to happen in the vicinity of residence, given that most happened in private residences |
| Location type | Living environment | 20 | Location type could be independent of event location, e.g., indoor/outdoor at the same location. However, it may depend on neighborhoods (e.g., a specific type does not exist in some neighborhoods) |
| Prior experience | multiple factors | 4,10,16,17 | All factors that affect overdose occurrence would also affect the chance of having prior experience |
| Poly-substance use,  Incarceration | Stress | 24, 25 | Stress from socioeconomic conditions may lead to substance abuse and related crime (Williams and Latkin, 2007) |

1. Sensitivity analysis results
   1. Impact of data exclusion

Supplementary table 2. Comparison between two identical models of fatal overdose risk, with (n = 35569) and without (n = 42711) filtering records with missing location information.

|  | Presented Model | | | Model without Filtering Data | | |
| --- | --- | --- | --- | --- | --- | --- |
| Variable | OR^1^ | 95% CI | p-value | OR^2^ | 95% CI | p-value |
| **Urbanicity** |  |  |  |  |  |  |
| Large | — | — |  | — | — |  |
| Medium | 1.27 | 1.10, 1.47 | 0.001 | 1.36 | 1.18, 1.56 | <0.001 |
| Small | 1.22 | 1.01, 1.47 | 0.035 | 1.35 | 1.13, 1.62 | 0.001 |
| Rural | 1.57 | 1.29, 1.90 | <0.001 | 1.62 | 1.34, 1.96 | <0.001 |
| **Recurrent Event** |  |  |  |  |  |  |
| No | — | — |  | — | — |  |
| Yes | 0.52 | 0.48, 0.56 | <0.001 | 0.52 | 0.48, 0.56 | <0.001 |
| **Age** |  |  |  |  |  |  |
| 18 - 29 | — | — |  | — | — |  |
| <18 | 0.50 | 0.36, 0.71 | <0.001 | 0.52 | 0.37, 0.72 | <0.001 |
| 30 - 49 | 1.73 | 1.59, 1.89 | <0.001 | 1.74 | 1.60, 1.89 | <0.001 |
| 50 - 64 | 2.71 | 2.45, 2.99 | <0.001 | 2.75 | 2.50, 3.03 | <0.001 |
| ≥65 | 2.76 | 2.37, 3.22 | <0.001 | 3.12 | 2.70, 3.61 | <0.001 |
| **Sex** |  |  |  |  |  |  |
| F | — | — |  | — | — |  |
| M | 1.62 | 1.50, 1.75 | <0.001 | 1.61 | 1.49, 1.73 | <0.001 |
| **Social Deprivation** |  |  |  |  |  |  |
| Q3 | — | — |  | — | — |  |
| Q1 | 1.28 | 1.12, 1.46 | <0.001 | 1.24 | 1.09, 1.41 | 0.001 |
| Q2 | 1.08 | 0.95, 1.22 | 0.2 | 1.07 | 0.95, 1.20 | 0.3 |
| Q4 | 0.99 | 0.88, 1.11 | 0.9 | 0.97 | 0.87, 1.09 | 0.6 |
| Q5 | 0.96 | 0.87, 1.06 | 0.4 | 0.94 | 0.85, 1.04 | 0.2 |
| **Benzodiazepines Prescription** |  |  |  |  |  |  |
| No | — | — |  | — | — |  |
| Yes | 1.43 | 1.26, 1.63 | <0.001 | 1.44 | 1.27, 1.63 | <0.001 |
|  |  |  |  |  |  |  |
| **Other Sedatives Prescription** | — | — |  | — | — |  |
| No | 0.89 | 0.81, 0.96 | 0.005 | 0.88 | 0.81, 0.95 | 0.002 |
| Yes |  |  |  |  |  |  |
| **Opioids for pain Prescription** | — | — |  | — | — |  |
| No | 1.14 | 1.00, 1.30 | 0.054 | 1.18 | 1.04, 1.34 | 0.009 |
| Yes | 1.27 | 1.10, 1.47 | 0.001 | 1.36 | 1.18, 1.56 | <0.001 |
| **Year** |  |  |  |  |  |  |
| 2015-2016 | — | — |  | — | — |  |
| 2017-2018 | 1.40 | 1.29, 1.52 | <0.001 | 1.47 | 1.36, 1.60 | <0.001 |
| **Urbanicity * Year** |  |  |  |  |  |  |
| Medium * 2017-2018 | 0.86 | 0.72, 1.03 | 0.11 | 0.80 | 0.67, 0.96 | 0.014 |
| Small * 2017-2018 | 1.07 | 0.86, 1.35 | 0.5 | 0.98 | 0.79, 1.22 | 0.9 |
| Rural * 2017-2018 | 0.89 | 0.70, 1.14 | 0.4 | 0.89 | 0.70, 1.12 | 0.3 |
| ^1^Adjusted for all covariates except location type | | | | | | |
| ^2^Adjusted for the same covariates including data with missing location type | | | | | | |

- 1. Comparison between spatial and non-spatial models

Supplementary table 3. Comparison between a conventional logistic regression model of fatal overdose risk and an equivalent Generalized Additive Model with spatial smoothing.

|  | Urbanicity Model | | | Spatial Smoothing Model | | |
| --- | --- | --- | --- | --- | --- | --- |
| Variable | OR^1^ | 95% CI^1^ | p-value | OR^1^ | 95% CI^1^ | p-value |
| **Urbanicity** |  |  |  |  |  |  |
| Large | — | — |  | — | — |  |
| Medium | 1.14 | 0.98, 1.32 | 0.090 |  |  |  |
| Small | 1.03 | 0.85, 1.24 | 0.8 |  |  |  |
| Rural | 1.31 | 1.07, 1.60 | 0.009 |  |  |  |
| **Location Type** |  |  |  |  |  |  |
| Private | — | — |  | — | — |  |
| Public Buildings | 0.08 | 0.06, 0.09 | <0.001 | 0.20 | 0.18, 0.22 | <0.001 |
| Healthcare Facilities | 0.19 | 0.14, 0.27 | <0.001 | 0.32 | 0.27, 0.37 | <0.001 |
| Outdoor | 0.16 | 0.15, 0.18 | <0.001 | 0.29 | 0.27, 0.31 | <0.001 |
| Others | 0.65 | 0.57, 0.75 | <0.001 | 0.68 | 0.62, 0.74 | <0.001 |
| **Recurrent Event** |  |  |  |  |  |  |
| No | — | — |  | — | — |  |
| Yes | 0.60 | 0.56, 0.65 | <0.001 | 1.5 | 1.5, 1.6 | <0.001 |
| **Age** |  |  |  |  |  |  |
| <30 | — | — |  | — | — |  |
| <18 | 0.54 | 0.38, 0.76 | <0.001 | 0.55 | 0.39, 0.78 | <0.001 |
| <50 | 1.68 | 1.54, 1.83 | <0.001 | 1.8 | 1.6, 1.9 | <0.001 |
| <65 | 2.30 | 2.07, 2.54 | <0.001 | 2.6 | 2.3, 2.8 | <0.001 |
| 65+ | 2.24 | 1.91, 2.64 | <0.001 | 2.6 | 2.2, 3.0 | <0.001 |
| **Sex** |  |  |  |  |  |  |
| F | — | — |  | — | — |  |
| M | 1.81 | 1.67, 1.96 | <0.001 | 1.8 | 1.6, 1.9 | <0.001 |
| **Social Deprivation** |  |  |  |  |  |  |
| Q3 | — | — |  | — | — |  |
| Q1 | 1.22 | 1.06, 1.40 | 0.004 | 1.3 | 1.1, 1.4 | 0.001 |
| Q2 | 1.12 | 0.99, 1.27 | 0.083 | 1.1 | 1.0, 1.3 | 0.075 |
| Q4 | 1.00 | 0.88, 1.12 | >0.9 | 1.0 | 0.89, 1.1 | 0.9 |
| Q5 | 1.00 | 0.90, 1.11 | >0.9 | 1.0 | 0.87, 1.1 | 0.5 |
| **Benzodiazepines Prescription** |  |  |  |  |  |  |
| No | — | — |  | — | — |  |
| Yes | 1.37 | 1.20, 1.56 | <0.001 | 1.4 | 1.2, 1.6 | <0.001 |
| **Other Sedatives Prescription** |  |  |  |  |  |  |
| No | — | — |  | — | — |  |
| Yes | 0.88 | 0.81, 0.96 | 0.004 | 0.86 | 0.79, 0.94 | 0.001 |
| **Opioids for pain Prescription** |  |  |  |  |  |  |
| No | — | — |  | — | — |  |
| Yes | 1.04 | 0.91, 1.19 | 0.6 | 1.1 | 1.0, 1.3 | 0.10 |
| **Year** |  |  |  |  |  |  |
| 2015-2016 | — | — |  |  |  |  |
| 2017-2018 | 1.39 | 1.28, 1.52 | <0.001 |  |  |  |
| **Urbanicity * Year** |  |  |  |  |  |  |
| Medium * 2017-2018 | 0.91 | 0.75, 1.09 | 0.3 |  |  |  |
| Small * 2017-2018 | 1.05 | 0.83, 1.33 | 0.7 |  |  |  |
| Rural * 2017-2018 | 0.90 | 0.70, 1.15 | 0.4 |  |  |  |
| ^1^OR = Odds Ratio, CI = Confidence Interval | | | | | | |

- 1. Alternative smoothers


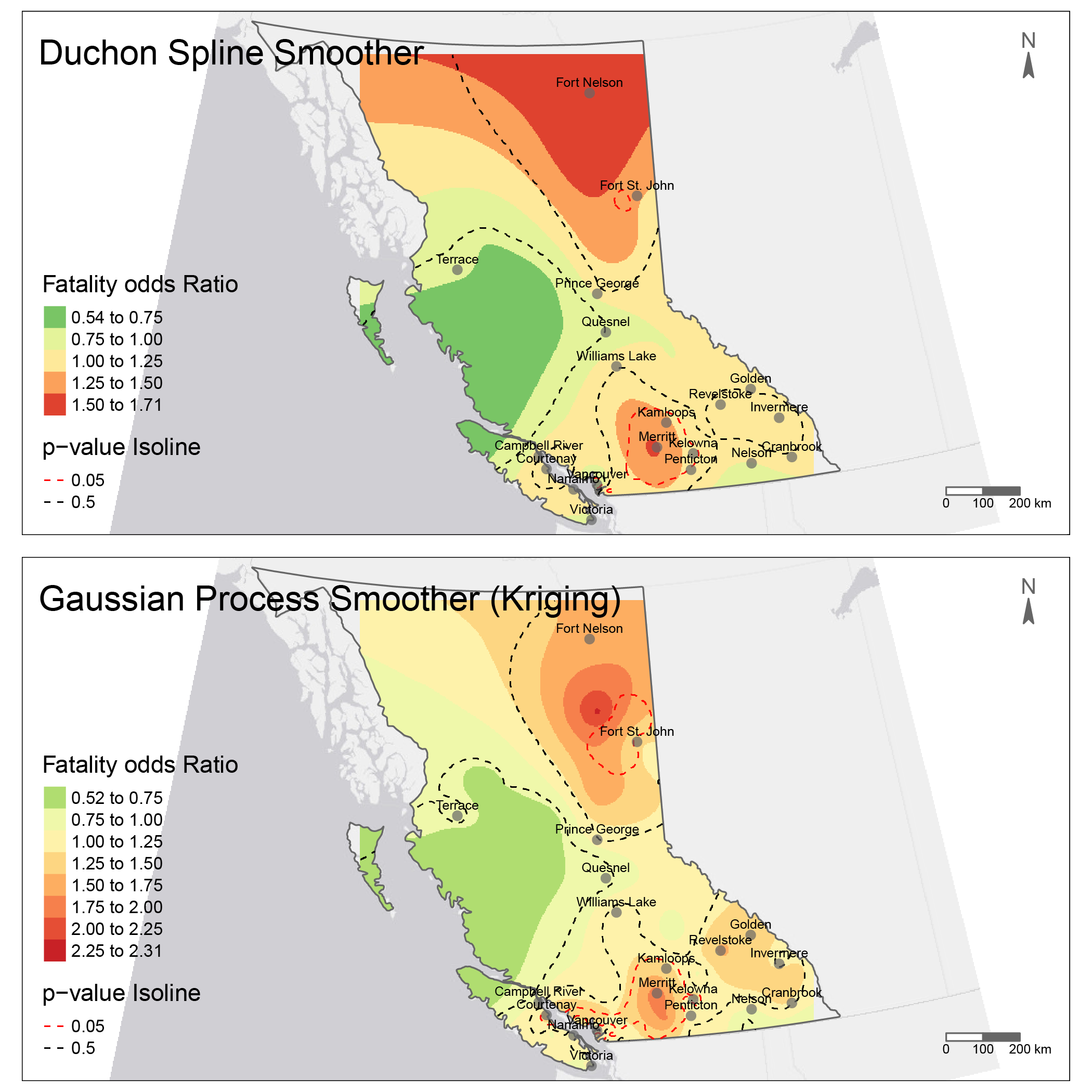


Supplementary figure 2. Comparison between two identical Generalized Additive Models of fatal overdose risk with different spatial smoothers.

1. Reference

Amlani, A., McKee, G., Khamis, N., Raghukumar, G., Tsang, E., Buxton, J.A., 2015. Why the FUSS (Fentanyl Urine Screen Study)? A cross-sectional survey to characterize an emerging threat to people who use drugs in British Columbia, Canada. Harm Reduct. J. 12, 1–7. https://doi.org/10.1186/s12954-015-0088-4

Bukten, A., Stavseth, M.R., Skurtveit, S., Tverdal, A., Strang, J., Clausen, T., 2017. High risk of overdose death following release from prison: variations in mortality during a 15-year observation period. Addiction 112, 1432–1439. https://doi.org/10.1111/add.13803

Kerr, T., Fairbairn, N., Tyndall, M., Marsh, D., Li, K., Montaner, J., Wood, E., 2007. Predictors of non-fatal overdose among a cohort of polysubstance-using injection drug users. Drug Alcohol Depend. 87, 39–45. https://doi.org/10.1016/j.drugalcdep.2006.07.009

Martins, S.S., Sampson, L., Cerdá, M., Galea, S., 2015. Worldwide prevalence and trends in unintentional drug overdose: A systematic review of the literature. Am. J. Public Health 105, e29–e49. https://doi.org/10.2105/AJPH.2015.302843

Pearl, J., 2009. Causality. Cambridge University Press, New York, UNITED STATES.

Rothman, K.J., Greenland, S., Lash, T.L., 2008. Modern epidemiology. Lippincott Williams \& Wilkins.

White, J.M., Irvine, R.J., 1999. Mechanisms of fatal opioid overdose. Addiction 94, 961–972. https://doi.org/10.1046/j.1360-0443.1999.9479612.x

Williams, C.T., Latkin, C.A., 2007. Neighborhood Socioeconomic Status, Personal Network Attributes, and Use of Heroin and Cocaine. Am. J. Prev. Med. 32, S203. https://doi.org/10.1016/J.AMEPRE.2007.02.006
